# Near-Basis-Set-Limit Double-Hybrid DFT Energies with Exceptionally Low Computational Costs

**DOI:** 10.1021/acs.jpclett.5c00122

**Published:** 2025-02-20

**Authors:** Dávid Mester, Mihály Kállay

**Affiliations:** † Department of Physical Chemistry and Materials Science, Faculty of Chemical Technology and Biotechnology, Budapest University of Technology and Economics, Műegyetem rkp. 3., H-1111 Budapest, Hungary; ‡ HUN-REN-BME Quantum Chemistry Research Group, Műegyetem rkp. 3., H-1111 Budapest, Hungary; ¶ MTA-BME Lendület Quantum Chemistry Research Group,Műegyetem rkp. 3., H-1111 Budapest, Hungary

## Abstract

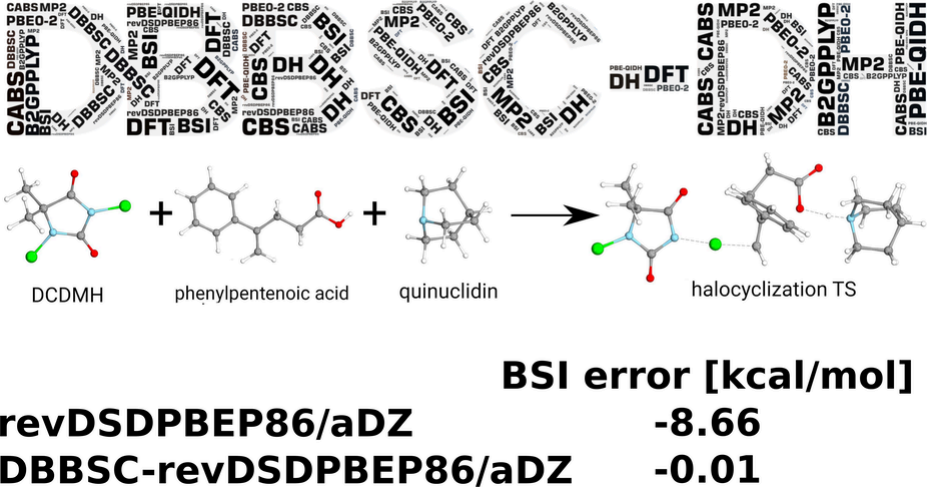

The use of density-based basis-set correction (DBBSC) [J. Phys. Chem. Lett.2019, 10, 293131090432 10.1021/acs.jpclett.9b01176] is extended to double-hybrid (DH) functionals. The proposed DBBSC-DH approach significantly reduces the basis-set requirements for accurate calculations, enabling near-basis-set-limit results using affordable one-electron basis sets. The accuracy of this method is comparable to that of the recently proposed DH functionals utilizing explicitly correlated (F12) second-order perturbation theory contribution [J. Phys. Chem. Lett.2022, 13, 933236178852 10.1021/acs.jpclett.2c02620PMC9575149]; however, its computational costs and resource demands are only a fraction of those associated with the DH-F12 scheme. Applications to real-life examples reveal that only a 30% overhead in wall-clock time is observed compared to conventional DH calculations, demonstrating that the DBBSC-DH approach is a compelling alternative to the excellent but relatively costly DH-F12 functionals, particularly for extended molecular systems.

Density functional theory (DFT) is regarded as one of the most powerful tools in quantum chemistry. Due to intensive method development over recent decades, it has evolved into the most widely used approach, which is unsurprising given its excellent cost-to-accuracy ratio.^1,2^ Among the more accurate approximations of the exact exchange-correlation (XC) functional are the double-hybrid (DH) functionals.^3^ These functionals combine DFT with wave function theory (WFT), wherein the semilocal DFT exchange and correlation are mixed with nonlocal Hartree–Fock (HF) exchange and second-order Møller–Plesset (MP2) correlation contributions. Thanks to modern hardware and implementations, the application of DH functionals to extended molecular systems no longer presents significant challenges when affordable one-electron basis sets are employed. However, due to the inherent properties of the theory, the slow basis set convergence of the MP2 correction—proportional to *L*^–3^, where *L* represents the highest angular momentum in the basis set—necessitates the use of extensive one-electron basis sets to approach the complete basis set (CBS) limit. This requirement significantly limits the size of systems that can be routinely investigated, although several attempts have been made to reduce the basis set demand by pairing DH functionals with tailored, small basis sets.^4−6^

To address basis set incompleteness (BSI) and simplify achieving CBS results, several approaches have been developed over past decades. Among the most notable ones are explicitly correlated (F12) theories,^7^ which improve convergence by introducing wave function ansätze that explicitly incorporate interelectronic distances. These approaches significantly reduce the basis set size required for results close to the CBS limit;^8,9^ however, at least with the same basis set size, the computation time is substantially increased, not to mention the excessive disk and memory usage. To reduce the basis set requirements of DH calculations, MP2-F12-based DH functionals (DH-F12) were first employed by Martin et al.^10,11^ Another approach to reducing wall-clock time relies on local approximations,^12−15^ which exploit the rapid decay of electron–electron interactions with distance. This trick leads to a significant reduction in wave function parameters and computational costs for extended systems. Relying on local MP2 (LMP2) calculations, the framework can be straightforwardly combined with DH theory (DH-LMP2),^16^ while MP2-F12 implementations using local approximations are also available.^17,18^ Such types of DH functionals have recently been introduced.^19^

An alternative approach for approximating CBS correlation energy is density-based basis set correction (DBBSC),^20,21^ proposed by Giner, Toulouse, and their co-workers. This method effectively combines WFT and DFT using a coordinate-dependent range-separation function. This function characterizes the spatial incompleteness of the one-electron basis set, and the missing short-range correlation effects are computed via a simple DFT energy correction. To improve the total energy, a correction to the HF energy is also required, which can be achieved using the complementary auxiliary basis set (CABS)^22^ correction known from F12 theory. The efficiency of this approach has been extensively demonstrated for pure WFT-based methods, such as MP2 and coupled-cluster singles and doubles with perturbative triples [CCSD(T)].^21,23,24^ Although it has been shown that the numerical results are very close, the correction-based approaches do not strictly outperform F12-based methods. Nevertheless, their extremely low computational cost and memory requirements make them particularly attractive, especially for large systems. Additionally, to further reduce costs and demands, the CABS and DBBSC calculations have been combined with local approximations and efficient grid prescreening techniques relying on our local natural orbital (LNO)-based CCSD(T) scheme.^25,26^ Using this framework, applying these corrections within DH-DFT to decrease basis set requirements for large molecular systems seems straightforward, but this has not yet been explored.

In this study, a procedure is presented for obtaining CBS-quality DH results at exceptionally low computational costs. In this scheme, the standard DH energies are improved using CABS and DBBSC corrections, while the efficiency of the approach for extended systems is demonstrated by exploiting local approximations. The performance and computational requirements are thoroughly compared with the competing F12-based DH functionals.

In DH theory,^3^ the first step involves performing a hybrid Kohn–Sham (KS) calculation, followed by adding an MP2-like second-order correction evaluated on the KS orbitals to the XC energy. The total electronic energy using a finite one-electron basis set  can be expressed as
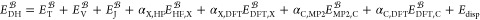
1where , , and  stand for the kinetic and electron–nuclear interaction energies, and the Coulomb self-interaction of the electron density, respectively.  and  correspond to the HF exchange and MP2 correlation energy, respectively. The terms  and  denote the exchange and correlation energy contributions from DFT, respectively, while *E*_disp_ is the functional-dependent dispersion correction.^27−29^ The mixing factors α control the relative contributions of the corresponding DFT, HF, and MP2 terms. In the more advanced spin-scaled DH functionals,^30−32^ the opposite-spin and same-spin contributions to  are scaled separately using different factors.^33^ This approach provides greater flexibility to the energy functional and enables a more accurate description of chemical properties. Building on this scheme, the definition of F12-based DH functionals becomes relatively straightforward.^11^ In this context, the MP2 contribution is replaced by the MP2-F12 contribution, while the CABS correction is also included.

In the proposed DBBSC-DH approach, the energy is improved through the incorporation of the CABS and DBBSC corrections. The approximation of the total energy in the CBS limit is calculated using the following simple form:
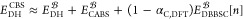
2where  and  represent the CABS and complementary density functional corrections, respectively, using the corresponding KS one-electron density *n*. It should be noted that the Fock matrix constructed in the CABS also includes the DFT contributions with the corresponding scaling factors. As a result, the DFT correlation energy is already improved, and accordingly, the DBBSC is scaled by (1 – α_C,DFT_) since it only needs to correct for the nonlocal correlation contribution. The theoretical background^20,34^ of this complementary DFT energy and its efficient implementation^23−25^ have been discussed extensively in the literature.

The performance of the methods was tested for popular DH functionals, including B2GPPLYP-D3-BJ,^35,36^ revDSDPBEP86-D4,^37^ PBE0-2-D4,^29,38^ and PBE-QIDH-D3-BJ.^39,40^ For simplicity, dispersion corrections will be omitted from the functional names in the following. This diverse selection is necessary because these functionals incorporate different mixing factors for the HF and MP2 contributions. The numerical results are first analyzed using the benchmark set introduced by Knizia, Adler, and Werner (KAW).^41^ Unlike in our previous works,^23,25,42^ we do not separate the compilation of atomization, open-shell, and closed-shell reaction energies into subsets. Instead, the results are discussed together to maintain the compactness of this letter. Detailed error metrics for the subsets are provided in the Supporting Information (SI), but in general, we can state that the conclusions drawn for the entire compilation are also valid for the subsets. The performance was extensively tested for various basis sets: the correlation consistent cc-pV*X*Z-F12 (*X* = D, T, Q)^43^ and aug-cc-pV*X*Z (*X* = D, T, Q)^44−48^ basis sets were employed. For the sake of brevity, the cc-pV*X*Z-F12 and aug-cc-pV*X*Z basis sets will be referred to as *X*Z-F12 and a*X*Z, respectively. The mean absolute errors (MAEs) with respect to the corresponding CBS values for each functional are shown in Figure 1.

**Figure 1:**
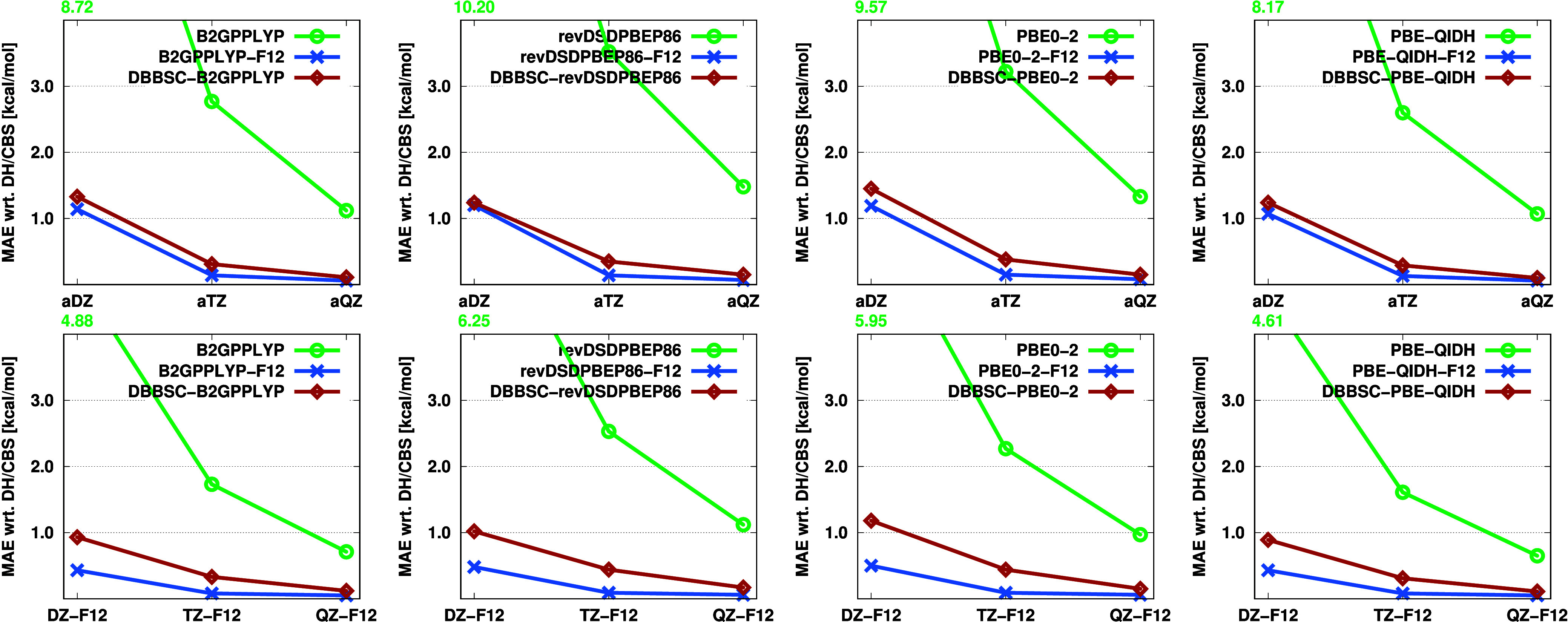
MAEs (in kcal/mol) for the KAW test set using standard, F12-, and DBBSC-DH functionals with various basis sets. For each functional, the corresponding CBS values were used as a reference.

The performance of the approaches and the observed trends are largely consistent across different functionals, with only minor variations. Let us first examine the results obtained with the a*X*Z basis sets. For the standard functionals, the MAE ranges between 8–10 kcal/mol using the aDZ basis set, while it drops below 1.5 kcal/mol with the DH-F12 and DBBSC-DH approaches. Increasing the basis set reduces the error for the standard methods to 2.5–3.5 kcal/mol, which still significantly exceeds chemical accuracy. In contrast, the errors decrease to 0.15 and 0.30 kcal/mol for the F12-based and DBBSC-DH functionals, respectively, firmly remaining below the threshold required for chemical accuracy. When comparing these two approaches, the differences align with our expectations,^23^ showing negligible variation. However, slightly better performance is observed with the DH-F12 methods. The largest difference, approximately 0.25 kcal/mol, is noted for the PBE0-2 functional, which is completely acceptable since this method contains the highest fraction of MP2 contribution.

Using the *X*Z-F12 basis sets, the errors are lower for all methods, particularly and unsurprisingly, for the DH-F12 approaches. For the standard functionals, the MAE is approximately 4.5–6.0 kcal/mol using the DZ-F12 basis sets, while for the DBBSC-DH methods, the error drops below 1 kcal/mol for all functionals except PBE0-2. Even better results were achieved with the F12-based approaches, although this advantage diminishes as the basis set increases, with monotonically decreasing errors.

The performance of DH approaches with respect to high-accuracy ab initio correlation methods is also an important measure of their practical applicability. To scrutinize this, the MAEs of the various DH functionals with respect to CCSD(T)/CBS reference values are depicted in Figure 2.

**Figure 2:**
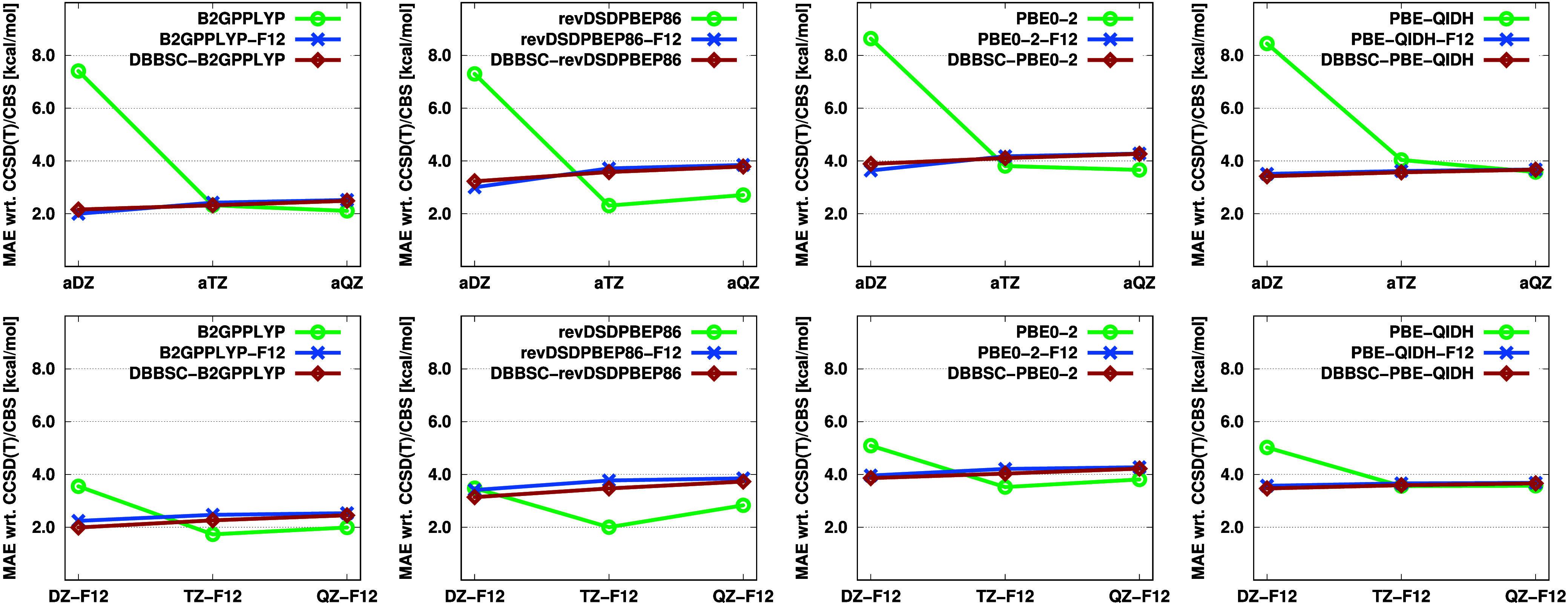
MAEs (in kcal/mol) for the KAW test set using standard, F12-, and DBBSC-DH functionals with various basis sets. For each functional, the CCSD(T)/CBS values were used as a reference. Dispersion corrections are included.

While a consistent trend in errors is observed here as well, more significant differences appear among the functionals. In general, it can be stated that the most accurate DH/CBS results were obtained with the B2GPPLYP functional, yielding an MAE of approximately 2 kcal/mol. This is followed by revDSDPBEP86 and PBE-QIDH, with errors around 3.5 kcal/mol, and the PBE0-2 functional, with an error of 4 kcal/mol. Examining the basis set dependence of the errors reveals that with the aDZ basis set, the DH-F12 and DBBSC-DH approaches are significantly more accurate than the standard functionals, with about 4–5 kcal/mol. As the basis set size increases, the trends are less straightforward to interpret, particularly for the revDSDPBEP86 and PBE0-2 functionals. In these cases, significant error cancellation occurs due to the opposing signs of the functional error and the BSI error. Nevertheless, it is clear that while the error for standard functionals varies significantly with increasing basis set size, it remains practically constant for the F12-based and DBBSC-DH functionals. The difference between these two approaches is negligible, with the largest deviations being 0.3, 0.1, and 0.04 kcal/mol for the aDZ, aTZ, and aQZ basis sets, respectively.

Similar trends are observed when applying the *X*Z-F12 basis sets, although the advantage of the DH-F12 and DBBSC-DH functionals diminishes slightly with the double-ζ basis set. In this case, the differences are around 2 kcal/mol for the B2GPPLYP and PBE-based functionals, while the errors are nearly identical for revDSDPBEP86. For the standard functionals, significant error cancellation still persists with increasing basis set size, whereas for the more advanced approaches, the MAE remains practically unchanged.

The efficiency of the DBBSC-DH approach was also examined for extended systems containing approximately 60–100 atoms by exploiting local approximations. The following discussion presents these results based on real-life applications taken from our previous works.^25,26^ The theoretical background of the local framework, the favorable error metrics arising from the approximations, and the details of the systems considered were thoroughly discussed in ref (25). First, barrier heights for a halocyclization^49,50^ and a Michael addition^26,51^ reaction are analyzed. Errors are presented with respect to high-quality DH-LMP2/CBS and LNO-CCSD(T)/CBS references. The numerical results are shown in Figure 3.

**Figure 3:**
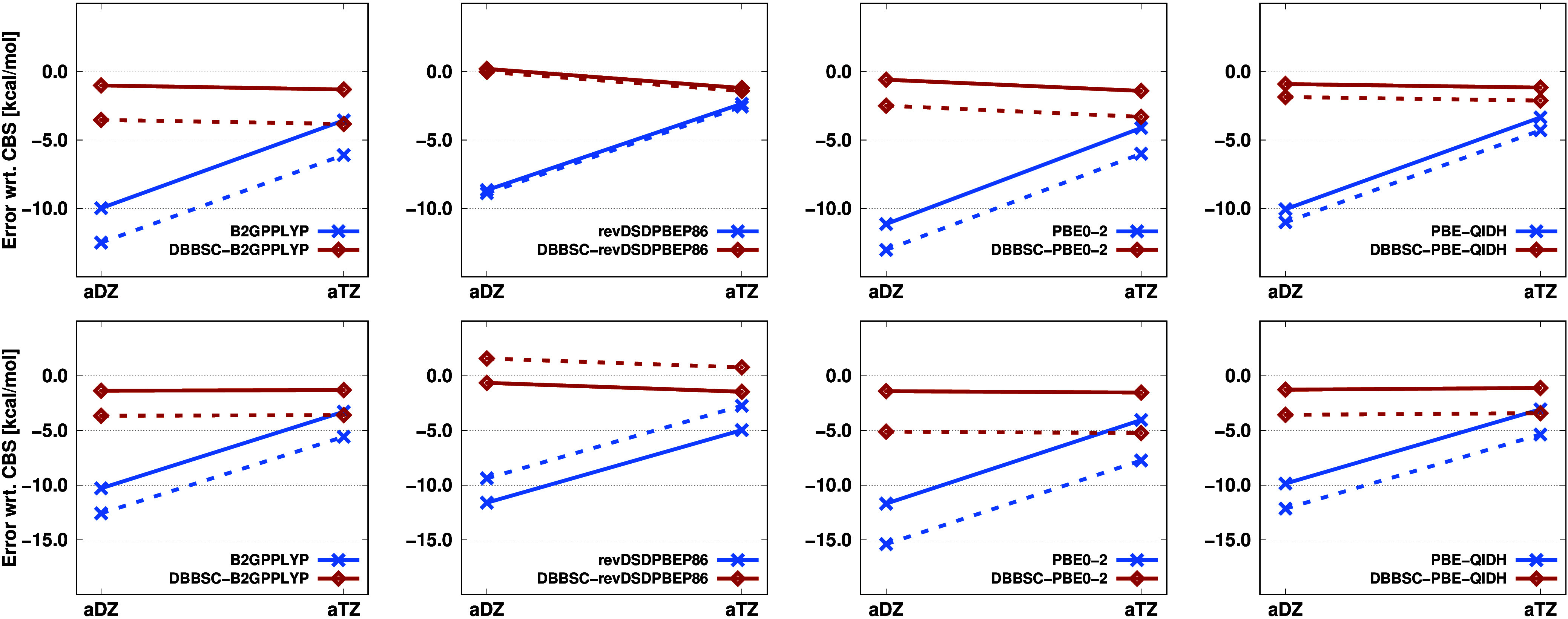
Errors (in kcal/mol) of the barrier heights for the halocyclization (top) and Michael addition (bottom) reactions using various functionals and basis sets. Errors relative to the DH-LMP2/CBS [LNO-CCSD(T)/CBS] references are depicted with solid (dashed) lines. The LNO-CCSD(T)/CBS reference for the halocyclization reaction (Michael addition) is 9.06 (−4.81) kcal/mol. Dispersion corrections are included.

For both reactions and all functionals, the application of the CABS and DBBSC corrections drastically reduces the BSI error. For the halocyclization reaction, using the aDZ basis set, the typical error of 8–11 kcal/mol for the standard functionals is reduced to below 1 kcal/mol, while significant improvements are observed for the aTZ basis set as well. For the Michael addition reaction, slightly higher errors are observed, but the improvements remain substantial. With the aDZ basis set, DBBSC-DH errors are below 1.5 kcal/mol in all cases relative to the DH-LMP2/CBS references.

One minor drawback is that the errors for the DBBSC-DH calculations do not consistently decrease with increasing basis set size, though this is hardly surprising given the nearly perfect results achieved with the double-ζ basis sets for both reactions. Compared to LNO-CCSD(T)/CBS references, the error of the method and the BSI error do not cancel each other, resulting in slightly increased errors for both the standard and the DBBSC-DH calculations. The most accurate results are provided by the DBBSC-revDSDPBEP86 functional, where the barrier height errors using the aDZ basis set are −0.23 and 1.33 kcal/mol for the halocyclization and Michael addition reactions, respectively.

The isomerization energies for two intermediate steps in a biosynthesis process (ISOL4)^26,52^ and the reaction energies for the AuAmin organometallic reaction^26,53^ were also studied. The results are presented in Figure 4.

**Figure 4:**
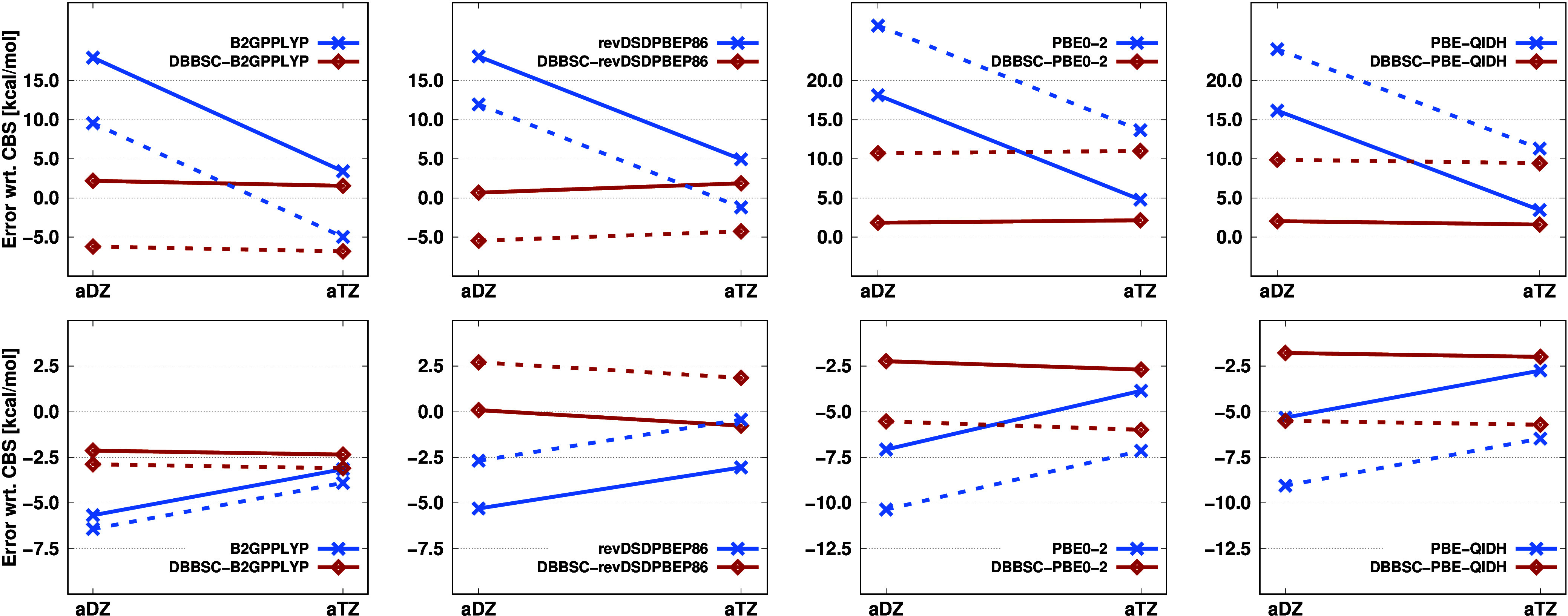
Errors (in kcal/mol) of the ISOL4 isomerization (top) and the AuAmin organometallic (bottom) reactions using various functionals and basis sets. Errors relative to the DH-LMP2/CBS [LNO-CCSD(T)/CBS] references are depicted with solid (dashed) lines. The LNO-CCSD(T)/CBS reference for the ISOL4 (AuAmin) reaction is 69.52 (−49.56) kcal/mol. Dispersion corrections are included.

As shown, the CABS and DBBSC corrections significantly accelerate approaching the DH-LMP2/CBS values. For the ISOL4 reaction, the error decreases from 16–18 to 1–2 kcal/mol using the aDZ basis set, while for the AuAmin reaction, it decreases from 5–7 to 2 kcal/mol. With the aTZ basis set, the results continue to improve compared to those obtained without corrections.

If the LNO-CCSD(T)/CBS values are used as references, the discussion becomes more complex due to the previously observed error compensation. For the ISOL4 reaction, when using the B2GPPLYP and revDSDPBEP86 functionals, the DH-LMP2 results slightly underestimate the LNO-CCSD(T) reaction energies, whereas with smaller basis sets, the DH-LMP2/CBS results are significantly overestimated. As the basis set size increases, the two errors compensate each other, yielding numerically accurate outcomes for these functionals even without the CABS and DBBSC corrections. Nevertheless, with the economical double-ζ basis set, the results are more accurate with the corrections. For the PBE0-2 and PBE-QIDH functionals, the DBBSC-DH results are consistently more accurate. For the AuAmin reaction, the BSI errors are approximately −5 kcal/mol in all cases when using the aDZ basis set. The DH-LMP2/CBS values are also lower than the LNO-CCSD(T)/CBS reference, except for revDSDPBEP86. Consequently, for the other functionals, the DBBSC-DH results are more favorable in all cases. However, for the revDSDPBEP86 functional, a smaller error is obtained with the standard functional when using the aTZ basis set due to favorable error compensation associated with the increasing basis set size and the milder BSI error. For both reactions, as in the previous examples, the most accurate results were achieved with the DBBSC-revDSDPBEP86 functional, where the reaction energy errors using the aDZ basis set are −5.11 and 2.52 kcal/mol for the ISOL4 and AuAmin reactions, respectively.

As a final example, the interaction energy of the notoriously complicated coronene dimer^54,55^ is discussed. The numerical results are depicted in Figure 5.

**Figure 5:**
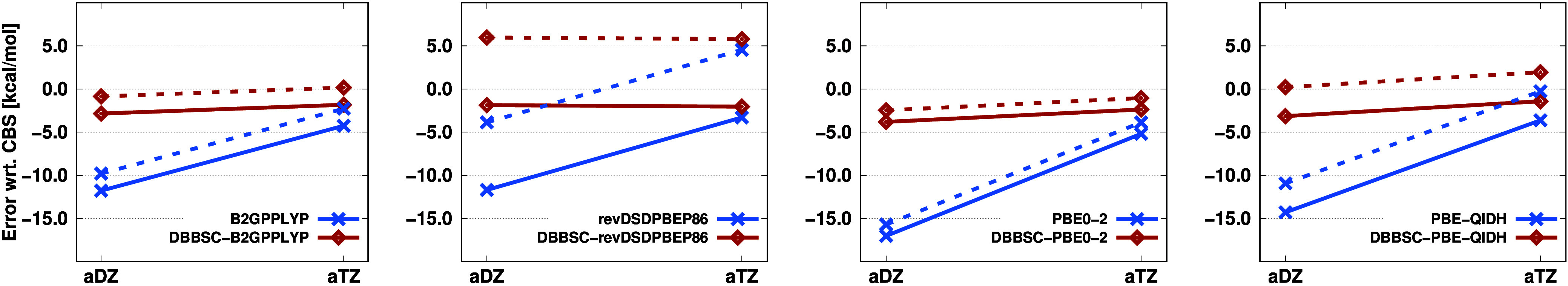
Errors (in kcal/mol) for the interaction energy of the coronene dimer using various functionals and basis sets. Errors relative to the DH-LMP2/CBS [LNO-CCSD(T)/CBS] references are depicted with solid (dashed) lines. The LNO-CCSD(T)/CBS reference is −25.60 kcal/mol. Dispersion corrections are included.

With the standard B2GPPLYP and revDSDPBEP86 functionals, the BSI error is approximately 12 kcal/mol using the aDZ basis set, while for the PBE0-2 and PBE-QIDH functionals, it is around 17 and 14 kcal/mol, respectively. The corrections reduce this error to 2–4 kcal/mol, significantly improving accuracy. Increasing the basis set size further reduces the error; the errors obtained for the standard functionals decrease from 3–5 to 1.5–2.5 kcal/mol.

Compared to the LNO-CCSD(T)/CBS reference and considering the DH-LMP2/CBS values, the magnitude of the interaction energy is underestimated by 8 kcal/mol for revDSDPBEP86, while the underestimation is only a few kcal/mol for the other functionals. As observed, the magnitudes of the interaction energies are significantly larger when smaller basis sets are used. Consequently, substantial error cancellation occurs for revDSDPBEP86, resulting in numerically accurate results even with double-ζ basis sets and without the CABS and DBBSC corrections. However, as the basis set size increases, the error for revDSDPBEP86 also increases, demonstrating the unreliability of the standard calculations. For the other functionals, significantly more accurate results are obtained with the DBBSC-DH calculations, while the robustness of the DBBSC-DH scheme is also clearly demonstrated as the errors remain practically constant. For this example, the most balanced results are achieved with the DBBSC-B2GPPLYP functional, with an error of −0.86 (0.17) kcal/mol using the aDZ (aTZ) basis set, although the performance of PBE-QIDH is also noteworthy.

When discussing the performance of methods, a particularly important aspect, aside from accuracy, is the computational cost of the approaches. In our previous study,^23^ we stated that DBBSC and MP2-F12 calculations take approximately the same amount of time when local approximations are not utilized. However, we now revise this claim for two reasons: (i) as demonstrated in ref (25), extremely small numerical quadrature is sufficient for accurate DBBSC calculations; (ii) a more efficient procedure for DBBSC has been implemented by reorganizing our algorithm. These improvements show that DBBSC calculations require significantly less effort than determining the MP2-F12 contributions. To demonstrate this, wall-clock times were measured for two molecules using the PBE0-2 functional: the 34-atom triphenylphosphine (PPh3) and the 98-atom reactant of the AuAmin reaction. For the latter, local approximations were employed for the post-KS steps. The results are summarized in Table 1.

**Table 1:** Wall-Clock Times (in min) Required for the Main Steps of DH Calculations for the PPh3 and AuAmin Molecules

		step					total		
Molecule	Basis set	KS	MP2	CABS	MP2-F12	DBBSC	DH	DH-F12	DBBSC-DH
PPh3	aDZ	6.09	3.06	2.98	14.48	0.25	9.15	23.55	12.39
	aTZ	14.09	15.13	7.21	44.96	0.69	29.22	66.26	37.12
AuAmin^a^	aDZ	95.11	74.19	34.31	–	38.93	169.29	–	242.53
	aTZ	270.79	320.14	83.88	–	95.47	590.94	–	770.28

a Local approximations were employed for the post-KS steps.

For PPh3, it can be observed that when using the aDZ basis set, solving the KS problem is more expensive than calculating the MP2 contributions. However, as the basis set size increases, the MP2 calculations become more dominant. This highlights the importance of minimizing basis set requirements for such calculations. The CABS correction, where the rate-determining step involves a Fock matrix construction in the extended basis, takes approximately half the wall-clock time needed to solve the KS equations. Furthermore, the MP2-F12 calculation is 3–4 times more expensive than a conventional MP2 energy calculation. In contrast, the wall-clock time required for DBBSC is practically negligible, taking less than 1 min even with the aTZ basis set. Consequently, DH-F12 calculations take approximately three times as long as conventional DH calculations, while the overhead for DBBSC-DH functionals is only around 30%.

Examining the larger example yields similar conclusions. Thanks to the use of local approximations, the MP2 contributions are less dominant, even for such extended systems. Their computational cost is comparable to that of solving the KS equations. Proportionally, the time required for the CABS correction further decreases when utilizing local approximations. With the aTZ basis set, the CABS correction takes only one-third of the time needed to determine the KS orbitals, while the DBBSC calculation requires a similar amount of time as the CABS step. Again, the overhead remains around 30–40%, which seems to be a reasonable trade-off given the performance achieved.

Additionally, we highlight the resource requirements of these methods concerning disk and memory usage. As is well-known and emphasized in refs (11 and 19), MP2-F12 implementations, even when employing local approximations, demand substantial resources. In contrast, DBBSC calculations, even for systems exceeding 1,000 atoms, require only a few additional gigabytes of main memory at most, without any data transfer from the hard disk.^25^ Considering these attractive wall-clock times and resource requirements, as well as the oustanding numerical performance of the approach, we can conclude that DBBSC-DH functionals are compelling alternatives to DH-F12 functionals,^11,19^ especially for extended molecular systems.

## Computational details

All calculations were performed using the latest version of the Mrcc suite of quantum chemical programs.^56,57^ The DH-F12 calculations were based on our MP2-F12 implementation, as described in ref (58). The DBBSC implementation is based on refs (21, 23), and (25). For the atomic orbital basis sets, the a*X*Z^44−48^ and *X*Z-F12^43^ basis sets were employed. In the case of the halocyclization and AuAmin reactions, the aug-cc-pV(*X*+d)Z basis sets were applied. For the CABS, the “OPTRI” bases of Yousaf and Peterson^59,60^ were selected. The density fitting approximation was utilized at both the HF/KS and post-HF/KS levels using the corresponding auxiliary basis sets,^61,62^ as in our previous works.^23,25,58^ The convergence threshold for HF/KS energy was set to 10^–6^ E_h_, while the root-mean-square change in the density matrix was set to 10^–7^. The frozen core approximation was employed in all post-HF/KS calculations. For DBBSC, the smallest Treutler–Ahlrichs (TA1) numerical quadrature^63^ was used together with the Log3 radial grid of Mura and Knowles.^64^ For the KS calculations, the default adaptive integration grid of the Mrcc package was used. The reported computation times are wall-clock times measured on an AMD EPYC 7763 processor using 8 cores with 2.45 GHz clock speed and 256 MB of L3 cache.

The reference CBS values were calculated similar to those in our previous studies.^23,25,58^ For the KAW benchmark set,^41^ the HF/KS energy was calculated using the a6Z basis sets, while the correlation energy was obtained with a two-point extrapolation^65^ from the a5Z and a6Z results. For the extended systems, a(Q,5)Z energies were utilized in all cases, except for the Michael addition reaction at the LNO-CCSD(T) level, where the a(T,Q)Z results were taken. For these large-scale applications, the final HF/KS energy was calculated using the extrapolation formula proposed by Martin and Karton,^66^ while the final correlation energy was obtained using the standard inverse cubic formula.^65^

## Supporting Information Available

The Supporting Information is available free of charge on the ACS Publications Web site. Calculated total, atomization, reaction, and interaction energies (ZIP). The Supporting Information is available free of charge at https://pubs.acs.org/doi/10.1021/acs.jpclett.5c00122.

(XLSX)
Transparent Peer Review report available (PDF)
