# Supplementary material for: Near-Basis-Set-Limit Double-Hybrid DFT Energies with Exceptionally Low Computational Costs
Source: J Phys Chem Lett. 2025 Feb 20;16(9):2136–43. doi: 10.1021/acs.jpclett.5c00122 (PMC11891966; doi:10.1021/acs.jpclett.5c00122)
Supplement: Supplementary file 2 — jz5c00122_si_002.pdf [file jz5c00122_si_002.pdf]

jz-2025-001225.R1

Name: Peer Review Information for "Near-basis-set-limit double-hybrid DFT energies with exceptionally low computational costs"

First Round of Reviewer Comments

Reviewer: 1

Comments to the Author

Mester and Kallay report the developement and test of the density-based basis-set correction (DBBSC) by Giner and Toulouse to fast and reduce the basis set convergence of double hybrids. They test it on some prototypical reactions probing for atomization, kinetics, isomerization and noncovalent interaction energy properties, that are somehow representative of the energy properties tested by extensive benchmark sets. They show that the correction compares with the F12 approach while conserving a low computational cost. The letter is clear, very well written, and to me, suitable for a possible publication in JPCL.

I express below a minor concern:

- the double hybrids tested here mix different fractions of MP2 correlation, i.e., 36% for B2GP-PLYP, 59/6% for DSD-PBEP86, 50% for PBE0-2 and 33% for PBE-QIDH, however, the authors never exploited this point, just arging that they are chosen for their popularity. Looking at Fig. 1, a comment would be welcome on how fast DBBSC-DH converges in reference to DH-D12 function of the fraction of nonlocal correlation.

Reviewer: 2

Comments to the Author

The letter by Mester and Kállay presents an interesting and useful version of small-basis set double hybrid techniques with low computational cost and high accuracy, which can compete with recently published MP2-F12 based double hybrids. Double hybrids are the most accurate density functionals, yet they suffer from the unfortunate basis-set dependence of the underlying MP2 component. This hinders their application to larger systems of actual chemical relevance. The approach presented herein offers a useful solution to this problem. While more benchmarking at a

larger scale is needed, the ideas and results presented here merit rapid publication and I fully support acceptance of this work.

There are only some very minor point that I recommend to adjust.

1) How the DH/CBS limit was reached is unclear. What exponents were used for the correlation-energy extrapolation? In this context it might also be worthwhile to look at Kraus' discussion on CBS extrapolations for DFT methods: J. Chem. Theory Comput. 2020, 16, 9, 5712.

2) Please provide more technical specifications for the wall-clock times. Were they all obtained on the same machine, while no other calculation was running? What were the specifications of that machine? It is common practice to include that information when timings are reported.

3) What were the SCF convergence criterion and the grid size? These should be reported too in the comp details.

4) The letter could be more precise regarding the cited literature:

a) On p. 4, Ref 27 (DSD-BLYP) is cited to acknowledge the first time spin component scaling has been combined with double hybrids. However, the first studies doing that was published by Head-Gordon: J Phys Chem A 2008, 112:2702 and J Chem Phys 2009, 131:174105. I suggest to cite these two papers in addition to Ref. 27.

b) For the sake of completeness, one could also mention attempts to pair conventional double hybrids with tailored, small basis sets, even though the idea to approach the CBS limit is lacking from those studies: J. Chem. Theory Comput. 2019, 15, 5, 2944, RSC Adv., 2021,11, 26073, and J. Phys. Chem. A 2024, 128, 31, 6581.

c) it is common to not only cite the underlying functional but also the paper that has presented its dispersion correction parametrisation. For the revDSD functional, the existing citation is sufficient, but these three citations are missing on p. 5 (1st line):

i) B2GPPLYP-D3-BJ: Phys. Chem. Chem. Phys., 2011, 13, 6670

ii) PBE0-2-D4: Ref. 26

iii) PBE-QIDH-D3-BJ: Phys. Chem. Chem. Phys. 2017, 19, 13481.

Reviewer: 3

Comments to the Author

Review of the paper ”Near-basis-set-limit double-hybrid DFT energies with exceptionally low computational costs”

General comments

The paper proposes an application to the density-based basis set correction (DBBSC) of Giner, Toulouse *et. al.* in the context of double hybrid (DH) DFT functionals. The DH are very popular as the percentage of MP2 correlation typically improves the quality of energy differences, while nevertheless introducing a stronger finite basis-set error (FBSE) typical to wave function theory (WFT). The authors also combines the DBBSC with complete auxiliary basis set (CABS) in order to correct for the FBSE of the Hartree-Fock exchange also present in the DH. The methods proposed here leads to, as expected, a strong improvement of the basis set convergence of the DH results. The authors observe that typically only a triple zeta quality is needed to obtain near complete basis set (CBS) convergence. The authors test this new approach on popular benchmarks and investigate the variability with four different types of DH functionals, leading sensibly to the same qualitative results.

Although the application proposed here is rather straightforward and do not contains explicitly new theoretical developments, it is nevertheless important for the theoretical chemist community as it can allow to broaden significantly the application of DH functionals. The paper is well written, concise and definitely deserves publication after some minor corrections being done.

Comments

1. When referring to the DBBSC in the abstract, it would be better to cite the first reference [J. Chem. Phys. 149, 194301 (2018)] where all the theory is presented in details. The paper cited by the authors refers to the first benchmark of the DBBSC on the G2 set.
2. Page 4, line 50. Regarding the statement ”the DBBSC contribution is scaled by the corresponding OS-MP2 mixing factor as the DBBSC provides a correction to the OS correlation energy”. I am not sure to understand or agree with the latter. It is correct to say that the DBBSC quantifies the incompleteness of the basis set via the local range separation parameter $\mu^{\mathrm{B}}(\mathbf{r})$ based on the OS interaction and on-top pair density. Nevertheless, it eventually uses range-separated DFT functionals which do not differentiate the correlation among SS and OS. Therefore, I do not see the justification for setting the same parameter for the OS-MP2 in spin-scaled DH, rather than setting it to one. Could the author give a clearer explanation for this ?

3. Could the author be more specific on what exact methodology they use for the DBBSC. I guess that they use the functional based on the HF-PBE scheme proposed in Ref. 18 together with the DF as proposed by the present authors in Ref. 20, but it would be clearer to state it explicitly for the sake of reproducibility of the results.
4. Page 4, line 54. While the "efficient implementation" of the DBBSC has been indeed discussed in the Refs. 20, 21, 22 when referring to its "theoretical background", I am afraid that these references are not really meaningful. Indeed, the main theoretical aspects of the DBBSC were already introduced in [J. Chem. Phys. 149, 194301 (2018)], and a more rigorous mathematical definition is given in [J. Chem. Phys. 156, 044113 (2022)]. Therefore I suggest that the authors either change accordingly the references or remove the part of the sentence referring to the discussion of the "theoretical background".

Author's Response to Peer Review Comments:

Senior Editor
The Journal of Physical Chemistry Letters

February 9, 2025

Dear Editor:

Thank you very much for your electronic mail of February 6 concerning our manuscript jz-2025-001225 and for the referees' reports. We greatly appreciate the referees' efforts to improve the quality of our paper. Please find enclosed the revised manuscript.

We have made the following changes to the manuscript and we give the following answers to the referees' comments.

Reviewer 1:

1. The double hybrids tested here mix different fractions of MP2 correlation, i.e., 36% for B2GP-PLYP, 59/6% for DSD-PBEP86, 50% for PBE0-2 and 33% for PBEQIDH, however, the authors never exploited this point, just arging that they are chosen for their popularity. Looking at Fig. 1, a comment would be welcome on how fast DBBSC-DH converges in reference to DH-D12 function of the fraction of nonlocal correlation.

Thank you for your comment. In the original draft, we mentioned that "This diverse selection is necessary because these functionals incorporate different mixing factors for the HF and

MP2 contributions". Regarding convergence in Fig. 1, we stated that "The largest difference [between the DH-F12 and DBBSC-DH results], approximately 0.25 kcal/mol, is noted for the PBE0-2 functional, which is completely acceptable since this method contains the highest fraction of MP2 contribution".

Reviewer 2:

1. How the DH/CBS limit was reached is unclear. What exponents were used for the correlation-energy extrapolation? In this context it might also be worthwhile to look at Kraus' discussion on CBS extrapolations for DFT methods: J. Chem. Theory Comput. 2020, 16, 9, 5712.
   Thank you for this remark. Detailed information on CBS references is provided at the end of the "Computational Details" section.
2. Please provide more technical specifications for the wall-clock times. Were they all obtained on the same machine, while no other calculation was running? What were the specifications of that machine? It is common practice to include that information when timings are reported.
   The calculations were performed on machines with the same configuration, and no other calculations were running on them. The wall-clock times were primarily influenced by the processor, as the amount of I/O operations from the hard disk was negligible. As mentioned in the original draft, the reported wall-clock times were measured on an AMD EPYC 7763 processor with 8 cores. We have supplemented this with information on the processor's clock speed and cache, which are 2.45 GHz and 256 MB of L3 cache, respectively.
3. What were the SCF convergence criterion and the grid size? These should be reported too in the comp details.
   The convergence threshold for SCF energy was set to 10^{-6} E_h, while the rootmean-square change in the density matrix was set to 10^{-7}. We have supplemented the manuscript with this information. Information regarding the grid was already provided in the original draft in the "Computational Details" section.
4. The letter could be more precise regarding the cited literature:
   (a) On p. 4, Ref 27 (DSD-BLYP) is cited to acknowledge the first time spin component scaling has been combined with double hybrids. However, the first studies doing that was published by Head-Gordon: J Phys Chem A 2008, 112:2702 and J Chem Phys 2009, 131:174105. I suggest to cite these two papers in addition to Ref. 27.
   (b) For the sake of completeness, one could also mention attempts to pair conventional double hybrids with tailored, small basis sets, even though the idea to approach the CBS limit is lacking from those studies: J. Chem. Theory Comput. 2019, 15, 5, 2944, RSC Adv., 2021,11, 26073, and J. Phys. Chem. A 2024, 128, 31, 6581.
   (c) It is common to not only cite the underlying functional but also the paper thathas presented its dispersion correction parametrisation. For the revDSD functional, the existing citation is sufficient, but these three citations are missing on p. 5 (1st line):
      i. B2GPPLYP-D3-BJ: Phys. Chem. Chem. Phys., 2011, 13, 6670
      ii. PBE0-2-D4: Ref. 26
      iii. PBE-QIDH-D3-BJ: Phys. Chem. Chem. Phys. 2017, 19, 13481.

Thank you for the comment regarding the references. We have made the suggested modifications in the manuscript accordingly.

Reviewer 3:

1. When referring to the DBBSC in the abstract, it would be better to cite the first reference [J. Chem. Phys. 149, 194301 (2018)] where all the theory is presented in details. The paper cited by the authors refers to the first benchmark of the DBBSC on the G2 set.

   Thank you for this remark. The current form of DBBSC, where the on-top pair density is approximated by its uniform electron gas version, was published in the JPC Letters paper. Since we used this approach, we would like to keep this reference in the abstract.

2. Page 4, line 50. Regarding the statement "the DBBSC contribution is scaled by the corresponding OS-MP2 mixing factor as the DBBSC provides a correction to the OS correlation energy". I am not sure to understand or agree with the latter. It is correct to say that the DBBSC quantifies the incompleteness of the basis set via the local range separation parameter $\mu^B(\mathbf{r})$ based on the OS interaction and on-top pair density. Nevertheless, it eventually uses range-separated DFT functionals which do not differentiate the correlation among SS and OS. Therefore, I do not see the justification for setting the same parameter for the OS-MP2 in spin-scaled DH, rather than setting it to one. Could the author give a clearer explanation for this?

   Thank you for bringing this to our attention. We completely agree with this comment; however, we would like to note that we also do not agree with setting the scaling factor to one, as the DFT correlation energy is already improved by the CABS correction. That being said, we do obtain a more rigorous theory by scaling the DBBSC with $(1-\alpha_{C,DFT})$. This, of course, only makes a difference in the case of spin-scaled functionals. Accordingly, we have recalculated the results obtained with revDSDPBEP86, but this does not affect our conclusions in any way. The revised manuscript now includes these updated results and theoretical considerations.

3. Could the author be more specific on what exact methodology they use for the DBBSC. I guess that they use the functional based on the HF-PBE scheme proposed in Ref. 18 together with the DF as proposed by the present authors in Ref. 20, but it would be clearer to state it explicitly for the sake of reproducibility of the results.

   Thank you for this comment. Indeed, we have used the above methodology. In the revised manuscript, we have emphasized this in the "Computational Details" section.

4. Page 4, line 54. While the "efficient implementation" of the DBBSC has been indeed discussed in the Refs. 20, 21, 22 when referring to its "theoretical background", I am afraid that these references are not really meaningful. Indeed, the main theoretical aspects of the DBBSC were already introduced in [J. Chem. Phys. 149, 194301 (2018)], and a more rigorous mathematical definition is given in [J. Chem. Phys. 156, 044113 (2022)]. Therefore I suggest that the authors either change accordingly the references or remove the part of the sentence referring to the discussion of the "theoretical background".

   We agree with this remark. To improve readability and clarity, we have made the suggested changes.

Please consider our paper for publication in the "Physical Insights into Quantum Phenomena and Function" section of the Journal of Physical Chemistry Letters Thank you for your effort.

Sincerely Yours,

Da´vid Mester
